# Association of selected polymorphisms in *GPX4, COMT, pre-miR-125a, pre-miR-10a,* and *pre-miR-323b* genes in Iranian women with idiopathic recurrent pregnancy loss: A case-control study

**DOI:** 10.18502/ijrm.v20i2.10503

**Published:** 2022-03-21

**Authors:** Sara Nemati Vahedi, Babak Kheirkhah, Ali Akbar Malekirad, Sayed Mostafa Hosseini

**Affiliations:** ^1^Department of Cellular and Molecular Biology, Faculty of Basic Sciences, Islamic Azad University Sirjan Branch, Sirjan, Iran.; ^2^Department of Microbiology, Faculty of Veterinary Medicine, Islamic Azad University Baft Branch, Baft, Iran.; ^3^Department of Biology, Faculty of Basic Sciences, Payame Noor University, Tehran, Iran.; ^4^Human Genetic Research Center, Baqiyatallah University of Medical Sciences, Tehran, Iran.; ^5^Department of Molecular Genetics, Baqiyatallah Hospital, Iran University of Medical Sciences, Tehran, Iran.

**Keywords:** SNaPshot, Single-nucleotide polymorphisms, Recurrent pregnancy loss, Genotypes.

## Abstract

**Background:**

Recurrent pregnancy loss (RPL) is a major concern among women worldwide. However, the exact mechanisms underlying miscarriage are not well understood. Recent evidence suggests that single nucleotide polymorphisms in various genes, especially miRNAs, may be responsible for RPL.

**Objective:**

We surveyed the association between polymorphisms in *pre-miR-125a, pre-miR-10a, pre-miR-323b, GPX4, *and* GPX4* in Iranian women with idiopathic RPL.

**Materials and Methods:**

DNA was extracted from blood samples of 116 women with idiopathic RPL and 89 healthy women as controls who had previously had at least two successful pregnancies. Polymerase chain reaction was used for the amplification of the genes. Genotype screening along with SNaPshot were performed to detect different polymorphisms. Finally, the polymorphisms and frequency of each genotype were compared between the two groups.

**Results:**

The frequencies of polymorphisms in *pre-miR-125a* (p < 0.001) and *pre-miR-10a* (p = 0.04) were calculated among the case and control groups, which showed a statistical difference (p < 0.05), indicating an association between these polymorphisms and the symptoms of RPL. The frequencies of polymorphisms of genotypes in *GPX4, COMT* and *pre-miR-323b* did not demonstrate any difference between the two groups. Also, the amount of alleles in *pre-miR-125a* and *pre-miR-10a* were significantly different (p < 0.001 and p = 0.02, respectively) and the dominant inheritance model was proposed.

**Conclusion:**

In conclusion, *pre-miR-125a* and *pre-miR-10a* can be associated with RPL in women. The SNaPshot technique is a valuable tool to evaluate possible associations between polymorphisms and health conditions.

## 1. Introduction

Recurrent pregnancy loss (RPL) is defined as “two or more abortions before 24 weeks of gestation” (1, 2). According to previous studies (3), multiple factors are involved in this condition, i.e. chromosomal abnormalities, genetic disorders, uterine structural abnormalities, immune diseases, thrombophilia, thyroid abnormalities, infectious diseases, high parental age, and lifestyle. However, the reason for more than 50% of miscarriages is still unknown (4). Among the factors affecting RPL, genes responsible for fetal growth and development have considerable roles.

Single nucleotide polymorphisms (SNPs) in several genes for proteins or fetal development are associated with RPL. Phospholipid hydroperoxide glutathione peroxidase (*GPX4*) is involved in glutathione synthesis (5) and may be associated with RPL in women; however, the exact mechanism is not clear. Catechol-O-methyl transferase protein (*COMT*) is now increasingly reported to have a role in pregnancy. In particular, recent reports have noted its expression in fetal membranes. This enzyme is also active both in the placenta and in the decidua (6). Reports have demonstrated that hypertension in pregnancy can reduce the placental *COMT* activity (7). Recent studies have shown that women with severe preeclampsia have reduced placental *COMT* protein expression (8). Besides, micro RNAs (miRs) are among the newly discovered genetic factors associated with RPL. miRs are small non-protein coding RNAs causing gene silencing at the post-transcription level. They target about one-third of human gene expressions and also play a critical role in various gestational events such as implantation, ectopic pregnancy, pre-term labor, pre-eclampsia, low birth weight, RPL, and anti-inflammatory responses. Recent investigations on women with RPL have shown that the overexpression of *miR-133a* can lead to a decline in human leukocyte antigen G (HLA-G) translation by joining to untranslated regions (UTR-3'). This protein has an essential role in the mother's immune system tolerating the fetus, and the down-regulation of this protein leads to RPL (9). *miR-125* is a group of miRs which consists of two subgroups: *miR-125a* and *miR-125b*. The expression of these miRs occurs in different organs like the stomach, liver, lungs, rectum, breast, prostate, and ovaries. Research on these organs suggests that the malignant process may be controlled by preventing gene expression. Embryo development may also be affected by low secretion of the mother's *miR-125a *polymorphism. Previous studies have also shown that women with RPL have down-regulation of 41 miRs and overexpression of four miRs (10). Although the critical role of *miR-125a* in RPL is unclear, recent evidence has proposed that its down-regulation may induce RPL in multiple ways (11).

By considering the wide range of miR activities in messaging processes, especially in the female genital system, studies on these regulatory RNAs seem to be necessary. The importance of miscarriage necessitates more comprehensive studies on the role of miRs in RPL. Therefore, the current study aimed to use the SNaPshot method to investigate the association between polymorphisms of *pre-miR-125a* (rs41275794)*, pre-miR-10a *(rs3809783)*, pre-miR-323b *(rs56103835)*, GPX4 *(rs4680)and* GPX4 *(rs713041)*,* and RPL in Iranian women with idiopathic recurrent miscarriage.

## 2. Materials and Methods 

### Sample collection 

In the present case-control study, 116 women with idiopathic RPL and 89 healthy women as controls who had previously had at least two successful pregnancies (a total of 205 individuals) were included and referred to Baqiyatallah Hospital (Tehran, Iran) for sampling during 2017-2019.

A diagnosis of idiopathic RPL was confirmed by the expert gynecologist after evaluation of hormonal levels, immunological and hematological deficiencies, karyotyping, and uterine anomalies. Uterine anomalies were evaluated through hysterosalpingography, sonhysterography and hysteroscopy techniques. Healthy controls visited the hospital for checkups and no abnormalities were found in their physical or laboratory examinations. They did not have any history of RPL or previous related medical diseases.

Inclusion criteria were: (i) women with idiopathic RPL; and (ii) who had undergone complete clinical and pathologic examinations. Cases who met the following criteria were excluded from the study: (i) history of autoimmune or hormonal diseases; (ii) chromosomal aneuploidies or genetic abnormalities; and/or (iii) immunological or hematological disorders.

### DNA extraction 

In the first examination, after an overnight fast, 5 ml peripheral blood samples were collected and placed in the EDTA-containing tubes. Then, the rapid genomic DNA extraction (RGDE) method was used to extract genomic DNA (12).

Subsequently, a Nanodrop ND-1000 spectrophotometer (Thermo Sci., Newington, NH, USA) was used to determine the quantity and quality of the extracted DNA. A fraction of each DNA sample was placed on 1.5% denaturing agarose gel stained with Red Safe and was then subjected to electrophoresis to assess the DNA quality. Following that, the isolated DNA was stored at -20 C until further analysis.

### Uniplex polymerase chain reaction (PCR) amplification

After DNA extraction, the functional SNPs *pre-miR-125a* (rs41275794), *pre-miR-10a* (rs3809783), *pre*-*miR-323b* (rs56103835), *GPX4* (rs4680) and *GPX4* (rs713041) were selected in each gene for genotyping based on published data and Primer-BLAST in the National Center for Biotechnology Information dbSNP database.

Oligo analyzer software was used to consider or predict the formation of dimer or secondary structures of primer. Primer sequences and their amplicon size are shown in table I. Gene runner software was used to design appropriate single-base extension (SBE) primers, as mentioned in table II. All individuals were analyzed for the presence of these polymorphisms (13).

### Multiplex PCR amplification

To conduct the multiplex PCR, the following chemicals were prepared: 1 μM of each primer, 0.17 mM of dNTPs, 1.5 mM of MgCl ${}_{2}$ , one unit of Taq DNA polymerase, and 20 ng of template DNA in a 20 μl volume flask containing 10 X PCR buffer. The three conditions applied to the thermal cycle were: 1) 10 min at 95 C; 2) 30 sec at 95 C for 35 cycles; 3) 30 sec at 60 C, one min at 72 C and a final extension of 10 min at 72 C. Then, 3 μl of PCR product was run in 12% polyacrylamide gel to check for the quality and yield of the multiplex PCR. Next, the excess dNTPs and primers were removed by treating the 3 μl of remaining PCR products with one unit (1 μl) of exonuclease I. Then, three units of shrimp alkaline phosphatase and two units of exonuclease I (ExoSAP/USB, Cleveland, OH, USA) were used to purify the remaining PCR products, which was followed by incubation for 15 min at 37 C and 15 min at 80 C to remove excess dNTPs and primers, and to deactivate the Taq enzyme (14).

### SNaPshot method

The SNaPshot assay applied five SBE primers which simultaneously annealed adjacent to SNP variant in our reaction. As shown in table II, poly (dC) tails of different lengths were attached to the SBE primers.

The SNaPshot solutions for multiplex PCR contained PCR product (1 μl), master mix SNaPshot (1 μl), each SBE primer (1 μl) and DDW (2 μl) in a final volume of 5 μl. The three thermal protocols applied to the SNaPshot were: two min at 96 C; 10 sec at 96 C; five sec at 50 C and 30 sec at 60 C. dNTPs were removed from the second PCR product by treating the remaining PCR products with three units (1 μl) of shrimp alkaline phosphatase and then the following two thermal protocols: 45 min at 37 C and 15 min at 75 C. An ABI PRISM 3130 Genetic analyzer was used to conduct capillary electrophoresis after which Gene Mapper idx, Ver 4.0 (Applied Biosystems, Foster City, CA, USA) was used to analyze the results (14).

**Table 1:** Primers sequences of PCR


**NCBI db SNP ID**	**Rs number**	**PCR primers**	**Amplicon size (bp)**	**Tm+**
*GPX4*	rs713041	F: 5'-GGACCTGCCCCACTATTTCT-3' R: 5'-CGCTGGATTTTCGGGTCTG-3'	137	60/57
*COMT*	rs4680	F: 5'-GAGGCTCATCACCATCGAG-3' R: 5'-GGCCTGGTGATAGTGGGTTT-3'	209	57/59
*miR-125a*	rs41275794	F: 5'-AGTGGATCCTCTGACTCCC-3' R: 5'-AGCCAGAGACAGAAAGACCA-3'	106	60/58
*miR-10a*	rs3809783	F: 5'-CGAAGAAGGCGCGGAAAGT-3' R: 5'-TCACCAGACTGTCCTCATTCA-3'	230	58/60
*miR-323b*	rs56103835	F: 5'-CTGTGCAGAAGATGCAGGAA-3' R: 5'-GGCATCAGGTCCAAGAAGAC-3'	157	58/58
Rs: Reference SNP, NCBI: National Center of Biotechnology, db: Database, PCR: Polymerase chain reaction, F: Forward primer, R: Reverse primer, Tm+: Melting temperature, SNP: Single nucleotide polymorphism				

**Table 2:** Sequences of primers used for single-base extension reaction


**SNP ID**	**NCBI number**	**PCR forward primers**	**Allele**	**Primer length (nt)**
*GPX4*	rs713041	TCATGAGTGCCGGTGGAAGGCTCC	T/A	24
*COMT*	rs4680	CCCCCCCCCCCCCCCCGGATGGTGGATTTCGCTGGC	G/A	36
*miR-125a*	rs41275794	CCCCCCCCCCCCTCTGTGTCTCTATTTCTGTC	G/A	32
*miR-10a*	rs3809783	CCTTTCCAGAAGAAAAAAA	A/T	19
*miR-323b*	rs56103835	CCCCCCCCCCCCCCCCAATGCTGCGAGCAGTGCCACCTCA	T/A	40
PCR: Polymerase chain reaction, SNP: Single nucleotide polymorphism, nt: Nucleotide				

### Ethical considerations

This case-control study was approved by the institutional review board and ethical consideration committee of Baqiyatallah University of Medical Sciences (Code: IR.BMSU.REC.1398.054). Written informed consent was obtained from all individuals.

### Statistical analysis

Statistical analysis was conducted using the SPSS v. 21 statistics software. Data were shown as percentages of the mean or allele frequency. Pearson's Chi-square test was used to calculate inter-group significance. The homozygous and heterozygous genotypes of each group were unified as carriers and the odds ratios (OR) and 95% confidence intervals (CI) were obtained. Statistical significance was set at p < 0.05.

## 3. Results 

**Figure 1:**
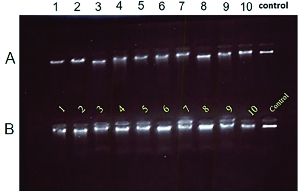
Quality of DNA extracted from peripheral blood samples, (A): The group of women with recurrent pregnancy loss, and (B): Control group. The control samples were positive DNA purified from samples.

The present study was performed on 116 cases with RPL and 89 healthy women without any history of RPL. There was no significant difference between their ages (38.93 ± 10.45 yr vs. 41.31 ± 10.74 yr, respectively; p = 0.40). After collection of blood samples, the DNA was extracted with the rapid genomic DNA extraction method, as shown in figure 1.

**Figure 2:**
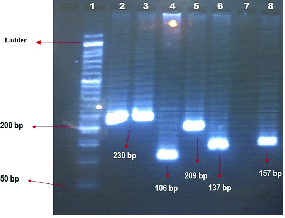
Unique polymerase chain reaction results, Lines 2 and 3: Product rs3809783 with a length of 230 bp, Line 4: Product rs41275794 with a length of 106 bp, Line 5: Product rs4680 with a length of 209 bp, Line 6: Product rs713041 with a length of 137 bp, Line 8: Product rs56103835 with a length of 157 bp, Line 7: Negative control, Line 1: Marker (50 bp DNA Ladder).

**Figure 3:**
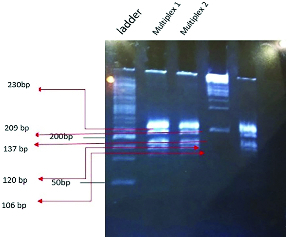
Multiplex PCR product, Ladder: Molecular size indicator (50 bp DNA Ladder), Multiplex 1: *GPX4* (rs713041) 137bp, *GPX4* (rs4680) 209bp, *miR-125a (*rs41275794) 106bp*, miR-10a *(rs3809783) 230bp*, miR-323b *(rs56103835) 120bp at 58 C, Multiplex 2: Repeated at 60 C.

The Uniplex and Multiplex PCR techniques were applied to detect G > A polymorphism in *pre-miR-125a* (rs41275794), A > T polymorphism in *pre-miR-10a* (rs3809783), T > A polymorphism in *pre-miR-323b* (rs56103835), A > G polymorphism in *GPX4* (rs4680) and C > T polymorphism in *GPX4* (rs713041) genes, as shown in figure 2 and figure 3, respectively.

**Figure 4:**
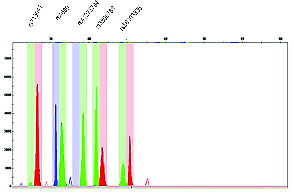
Electrophoresis results obtained from Gene Mapper idx version 4 software. SNaPshot multiplex analysis was performed to determine the sample-related genotyping with RPL symptoms, A (Green), T (Red), and G (Blue). The results showed that the cases were rs713041, rs41275794 locus homozygous and rs3809783, rs4680, rs56103835 locus heterozygous.

After multiplex PCR, the SNaPshot kit and specific SBE primers were used to detect rs713041, rs4680, rs41275794, rs3809783, and rs56103835 SNPs with the genetic analyzer machine, as shown in figure 4.

The comparison of SNP genotype distribution for all of the studied genes in the control and case groups is summarized in table III. The frequencies of AG, GG and AA genotypes in the polymorphism rs41275794 of *pre-miR-125a* (p < 0.001) and of TT, AT and AA genotypes in the polymorphism rs3809783of* pre-miR-10a* (p = 0.04) were calculated for the women in the case and control groups. The findings showed significant differences and an association of these polymorphisms with the symptoms of recurrent miscarriage (p < 0.05). There was no significant difference (p = 0.09) in the frequency of TT, AT and AA genotypes in the* GPX4* gene (rs713041 polymorphism) between the case and control groups. The frequency of TT, AT and AA genotypes in the *miR-323b* gene (rs56103835 polymorphism) showed no significant difference (p = 0.14) between the case and control groups. Also, no significant difference (p = 0.21) was observed in the frequency of GG, AG or AA genotypes in the* COMT* gene (rs4680 polymorphism) between the two groups (Table III). In the allele frequency study for alleles A and G of polymorphism rs41275794, a significant difference was found between the case and control groups (p < 0.001). Similarly, there was a significant difference (p = 0.02) in the frequency of alleles A and T of polymorphism rs3809783 between the two groups of cases and controls (Table IV). The assessment of risk at a 95% confidence level for allele A relative to G in the rs41275794 polymorphism showed that by changing the G allele to A, the risk of the RPL phenotype was increased by 1.912 times. Similarly, for the rs3809783 polymorphism, the changing of allele A to T increased the risk of the RPL phenotype by 1.704 times, showing a greater association of risk. To evaluate the genotypic inheritance of significant polymorphisms, we studied and calculated the risk of RPL in three models: dominant, recessive, and obvious models. The results showed that the dominant hereditary model had a significant correlation based on the genotypic frequency of the variant (Table V).

**Table 3:** Genotype frequencies of RPL and control groups


**Polymorphism**	**Genotypes**	**Cases (n = 116)**	**Control (n = 89)**	**p-value**
**rs41275794 G > A**	GG	46 (39.7)	54 (60.7)	< 0.001
	GA	59 (50.9)	31 (34.8)	
	**rs713041 T > A**	AA	11 (9.4)		4 (4.5)
TT		74 (63.8)	68 (76.4)	0.09
AT		31 (26.7)	18 (20.2)	
**rs56103835 T > A**		AA	11 (9.5)		3 (3.4)
	TT	60 (51.7)	55 (61.8)	0.14
	AT	45 (38.8)	31 (34.8)	
	AA	11 (9.5)	3 (3.4)	
**rs4680 G > A**	GG	62 (53.4)	58 (65.2)	0.21
	GA	45 (38.8)	27 (30.3)	
	AA	9 (7.8)	4 (4.5)	
**rs3809783 A > T**	AA	53 (45.7)	56 (62.9)	0.04
	AT	56 (48.3)	30 (33.7)	
	TT	7 (6.0)	3 (3.4)	
Data presented as n (%). Chi-square test was used. P-value < 0.05 was considered statistically significant				

**Table 4:** Allele frequency of RPL and control groups


**Polymorphism**	**Allele**	**Cases **	**Control**	**95% CI**		**OR**	**p-value**
				**Lower**	**Upper**		
**rs41275794 G > A**	G	151 (65.1)	139 (78.1)	1.224	2.987	1.912	< 0.001
	A	81 (34.9)	39 (21.9)				
**rs3809783 A > T**	A	162 (69.8)	142 (79.8)	1.075	2.702	1.704	0.02
	T	70 (30.2)	36 (20.2)				
Data presented as n (%). Chi-square test was used. OR: Odds ratio, CI: Confidence interval. P-value < 0.05 was considered statistically significant							

**Table 5:** Genotype inheritance


**Polymorphism**	**Genotypes (dominant)**	**Cases**	**Control**	**p-value**
**rs41275794 G > A**	GG	46 (39.7)	54 (60.7)	< 0.001
	AA+AG	70 (60.3)	35 (39.3)	
**rs3809783 A > T**	AA	53 (45.7)	56 (62.9)	0.01
	TT+AT	63 (54.3)	33 (37.1)	
Chi-square test was used. P-value < 0.05 was considered statistically significant				

## 4. Discussion

RPL is a clinical challenge that affects approximately 1-5% of women of childbearing age (11). The underlying mechanism of RPL in 37-79% of cases is unclear. Recent evidence has revealed that molecular and genetic disorders, including various polymorphisms, may be the main cause of RPL in these cases (15).

In this research, we compared for the first time five polymorphisms of *pre-miR-125a* (rs41275794), *pre-miR-10a* (rs3809783), *pre-miR-323b* (rs56103835), *GPX4* (rs4680) and *GPX4* (rs713041) between healthy women and women with RPL using the SNaPshot genotyping method. Our findings showed a significant difference in the *miR-125a* gene polymorphism rs41275794 between the control and case groups. GG was found to be the most common genotype in the control subjects (60.7%), while GA (50.9%) was the most common genotype in the case group. These data indicated that the polymorphism in *miR-125a* may be a risk factor for RPL among the Iranian female population. In our study, allele A was significantly associated with an increased risk of RPL indicating that this variant allele in *pre-miR125a* can change precursor and mature *miR-125a* expression.

Our data revealed that the dominant genotype was significantly associated with an increased risk of RPL. In another study, expression of mature *miR-125a* in the CC/AA genotype was ∼ 2.1-fold higher than that in the TT/AA genotype (16). They found that the A > T haplotype in rs41275794 affected *miR-125a* expression and caused RPL in women. In another study, Li and colleagues compared the expression pattern of six miRs, including *miR-125a, miR-125b, miR-34a, miR-155*, *miR-24* and *miR-141* between healthy women and those with RPL. They found that *miR-125a*, *miR-125b,* *miR-34a, miR-155 *and *miR-141* were significantly overexpressed in women with RPL while *miR-24* was significantly down-regulated in these cases, compared to the healthy group. Additionally, they showed that the A > T polymorphism in *pri-miR-10a* (rs3809783) was significantly associated with an increased risk of RPL in women.

More studies are being conducted nowadays to find relationships between SNPs and the miRNA system's critical components, biological functions and disease (4, 16, 17). The current study indicated that Iranian women with rs3809783 A > T in *pri-miR-10a* were significantly associated with an increased risk of RPL. This study's findings also suggested that the expression level of the *miR-10a* target gene, *Bim*, might have been reduced because of *miR-10a* rs3809783. Additionally, this study showed a significant correlation between the *miR-10a* rs3809783 polymorphism and the risk of RPL. AT was the most common genotype in the case group while the control group most commonly exhibited the AA genotype. The frequency of the T allele in the case group (30.2%) was significantly greater than that of the control group (20.2%). On the other hand, the control group had a significantly higher frequency (79.8%) of the A allele compared to the case group (69.8%).

The current study also found a significant association between the dominant genotype and an increased risk of RPL. Our data showed that polymorphisms in *miR-125a *and* miR-10a* can be associated with the risk of RPL in women; however, further studies are needed to confirm this. Several studies have considered the role of different miRs in RPL (17, 18). In a case-control study among Korean women, RPL cases had significantly different frequencies of the *miR-499TC* + CC and *miR-196a*2CC genotypes than the control group. It was shown that these two polymorphisms and their combinations were significantly associated with the incidence of idiopathic RPL in the study population (19). In addition, another study conducted in north Indian females showed that *miR-196a*2CC, *miR-499*TC + CC, and *miR-196a*2CC/*miR-499*TC + CC were associated with an increased risk of RPL (20).

Furthermore, cumulative gene risk score analyses have been calculated regarding oxidative stress-related genes, which suggested that common variations in individual genes like *GPX4* and to a greater extent in the multi-locus model (*ABCB1*, *GPX4*, *GPX4*, *OGG1* genes) are associated with RPL (21). In the current study, we did not find a significant difference in *miR-323b* and *GPX4* polymorphisms between the healthy and case groups. Previous studies have reported that SNPs in the* GPX4* gene significantly affect enzyme activity (6, 22, 23). The evidence shows that pre-eclampsia pathogenesis is associated with SNPs in this gene (6). A recent cohort study on a Korean population of 164 women with preeclampsia and 182 normotensive cases showed that preeclampsia was associated with the Val108/158Met polymorphism (6). Nevertheless, the variation in *GPX4* enzyme activity is likely not due to the Val108/158Met polymorphism alone. Additionally, various vascular health impairments can be due to reduced levels of estrogen metabolites, such as 2-methoxyestradiol, because of a decrease in *GPX4* activity (6, 24). Hypoxia-inducible factor-1a (HIF-1a) plays an essential role in angiogenesis and studies have found that this factor can be suppressed by anti-angiogenic 2-methoxyestradiol (6). Additionally, this transcription factor can induce genes such as soluble fms-like tyrosine kinase-1 (*sFlt-1*) which facilitates the adaptation and survival of cells during low-oxygen levels. Studies have also shown the stimulation of trophoblast invasion due to the combination of 2-methoxyestradiol and low levels of oxygen (25-27). Placental pathology can also be affected throughout the different stages of pregnancy if the homeostasis and regulation of *GPX4* and 2-methoxyestradiol are disrupted. Also, it has been hypothesized that hypoxia-driven trophoblast invasion and vascular remodeling can be disrupted with a premature increase in 2-methoxyestradiol, thereby contributing to preeclampsia pathogenesis (6, 25). Decreased *GPX4* activity in late pregnancy can cause reduced levels of 2-methoxyestradiol and thus reduce *HIF-1a* inhibition, resulting in possible vascular pathology and inflammation (6). Therefore, future studies on the expression and activity of the *GPX4* enzyme throughout pregnancy can pinpoint the effect of *GPX4*/2-methoxyestradiol at different stages of miscarriage.

Since there is no similar report on the frequency of selected polymorphisms in the Iranian female population, one of the aims of the current study was to investigate the possible role of these polymorphisms in the occurrence of spontaneous miscarriage. A strength of this study was the careful selection of eligible individuals within the case group, i.e. women who had had at least two recurrent miscarriages, were completely healthy in terms of anatomical and cytogenetic studies and did not have any significant clinical problems in their medical records. The findings indicated that genetic polymorphisms can influence RPL, but the lack of significant differences in the *GPX4, COMT *and *miR-323b* polymorphisms in the Iranian females also reinforced the hypothesis that a single miR or polymorphism is unlikely to be solely responsible for RPL. One of the limitations of our study was that there is high diversity of ethnicity in the Iranian population and of the individuals who participated in the investigation but there is a lack of indigenous genetic databases which contain allele frequencies and other genomic data for estimation of, for example, samples collection.

It is recommended that the expression of these polymorphisms in the blood as well as in the uterine tissue of women with RPL be measured to create a better understanding of the molecular mechanisms leading to miscarriage and to compare different conditions. Moreover, this research can be utilized to provide a clearer picture of the mechanisms involved in causing a miscarriage in women and to analyze a complete profile of polymorphisms involved in the various pathways leading to miscarriage.

Bioinformatics studies on the secondary and tertiary protein structures of selected genes using related databases can provide more comprehensive information on recurrent miscarriages.

## 5. Conclusion

In conclusion, our data illustrated that a polymorphism in *pre-miR-125a* (rs41275794) and in *pre-miR-10a* (rs3809783) can be associated with RPL in women. SNaPshot was shown to be a suitable method for investigation of the role of polymorphisms in the incidence of health conditions.

##  Conflict of Interest

The authors declare that there is no conflict of interest.
